# Supplementary figures and images for: The genes significantly associated with an improved prognosis and long-term survival of glioblastoma
Source: PLoS One. 2023 Nov 29;18(11):e0295061. doi: 10.1371/journal.pone.0295061 (PMC10686432; doi:10.1371/journal.pone.0295061)

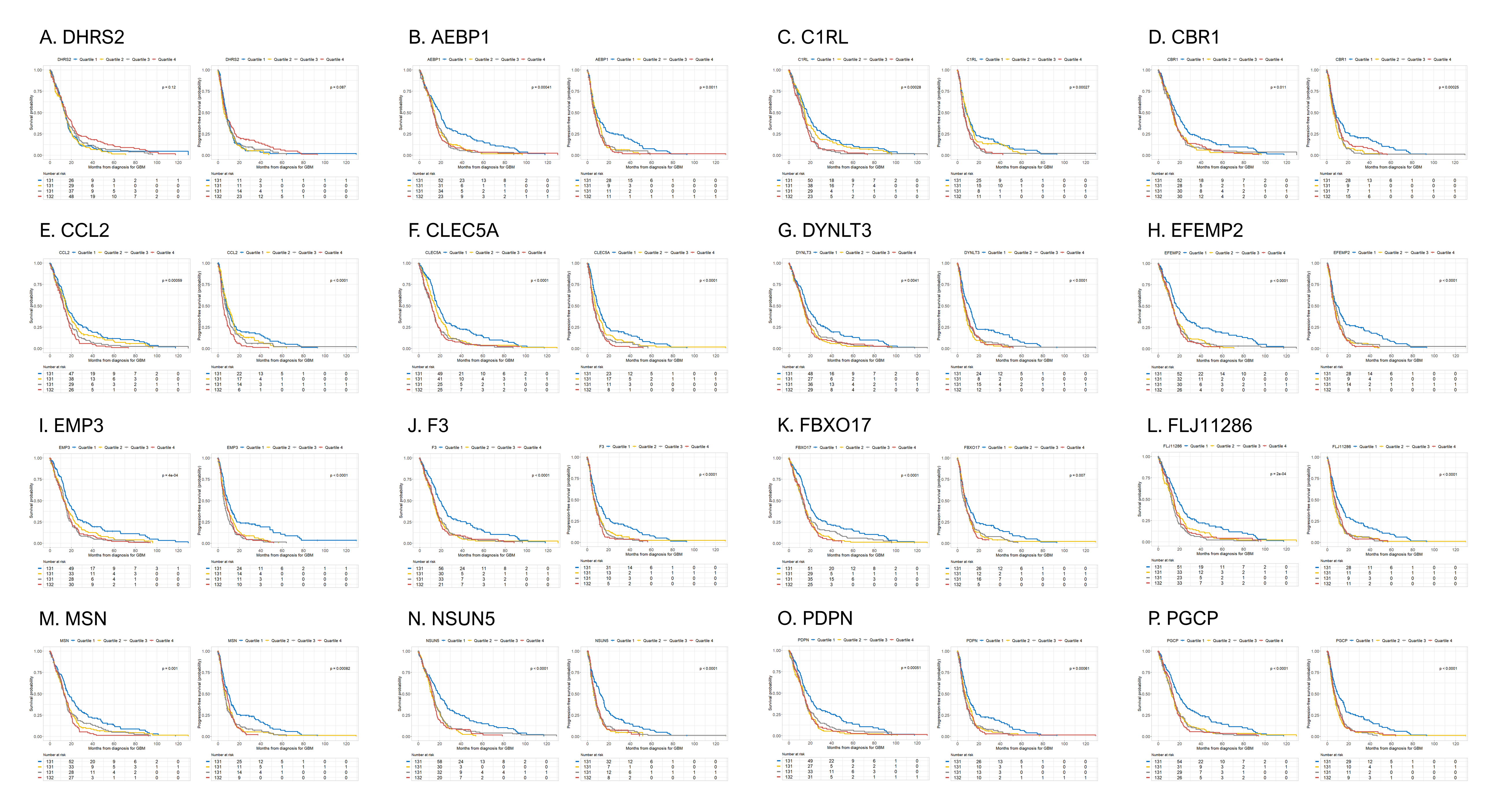

Supplement: S1 Fig — (TIF) [file pone.0295061.s002.tif]

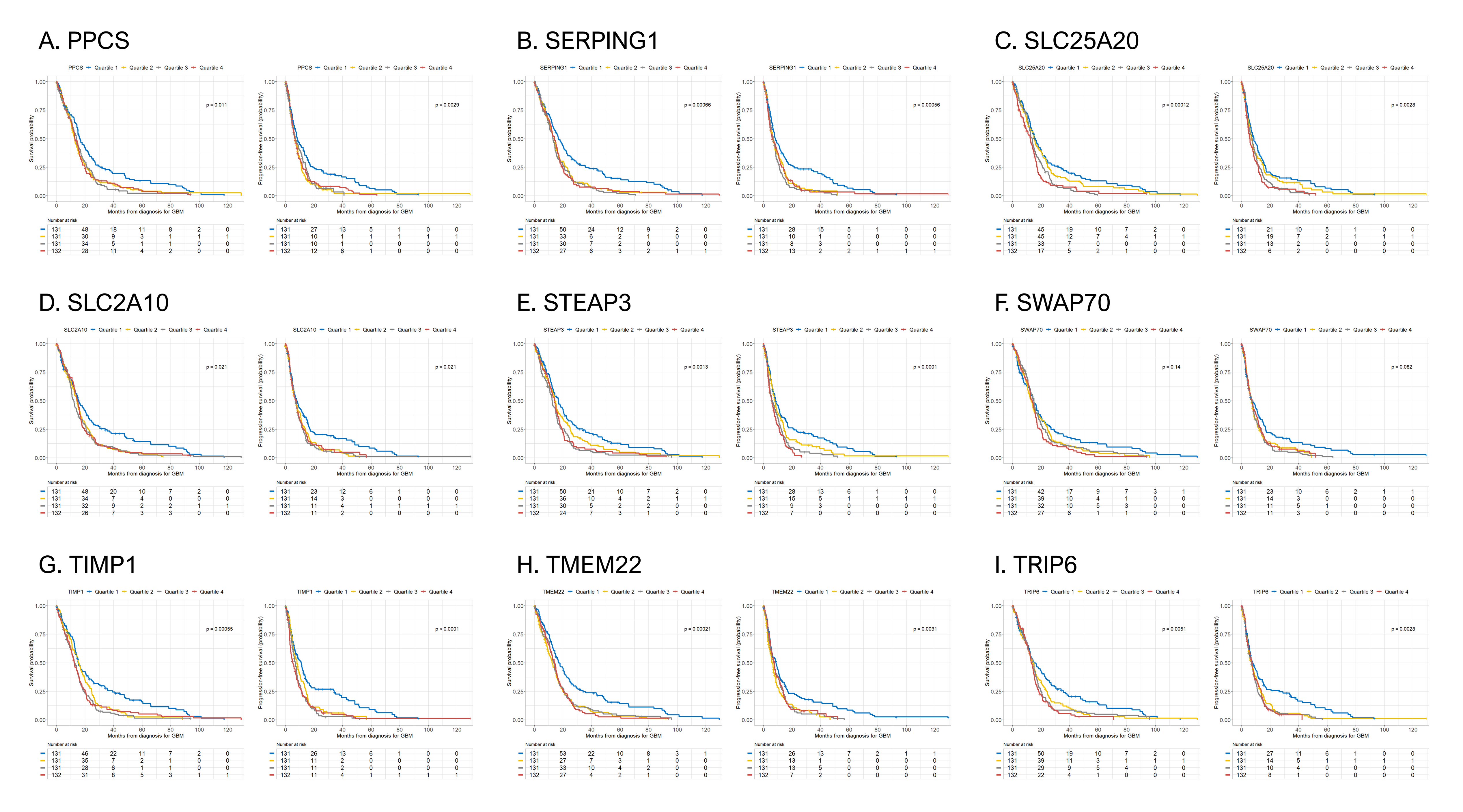

Supplement: S2 Fig — (TIF) [file pone.0295061.s003.tif]
