# Supplementary material for: The genes significantly associated with an improved prognosis and long-term survival of glioblastoma
Source: PLoS One. 2023 Nov 29;18(11):e0295061. doi: 10.1371/journal.pone.0295061 (PMC10686432; doi:10.1371/journal.pone.0295061)
Supplement: S1 Table — (DOCX) [file pone.0295061.s004.docx]

| Characteristics | Total |
| --- | --- |
| Number | 525 |
| Sex, female, n (%) | 205 (39.0) |
| Age at diagnosis of GBM, mean ± SD, y | 57.7 ± 14.6 |
| Time duration between GBM diagnosis and death (days), mean ± SD | 508.9 ± 539.4 |
| Time duration between GBM diagnosis and disease progression (days), mean ± SD | 307.0 ± 391.0 |
| Karnofsky performance scale score, median (IQR) | 80.0 (70.0–80.0) |
| Missing data, n (%) | 133 (25.3) |
| Radiation treatment, n (%) |  |
| Yes | 435 (82.9) |
| No | 70 (13.3) |
| Missing data | 20 (3.8) |
| Immune cells (CIBERSORT fraction), mean ± SD |  |
| CD8+ T cells | 0.022 ± 0.039 |
| Regulatory T cells | 0.010 ± 0.022 |
| Naive CD4+ T cells | 0.006 ± 0.021 |
| Resting CD4+ memory T cells | 0.082 ± 0.067 |
| Activated CD4+ memory T cells | 0.003 ± 0.011 |
| Memory B cells | 0.031 ± 0.040 |
| Plasma B cells | 0.017 ± 0.039 |
| Activated dendritic cells | 0.016 ± 0.022 |
| IQR, interquartile range; GBM, glioblastoma multiforme; SD, standard deviation | |
