# Supplementary material for: The genes significantly associated with an improved prognosis and long-term survival of glioblastoma
Source: PLoS One. 2023 Nov 29;18(11):e0295061. doi: 10.1371/journal.pone.0295061 (PMC10686432; doi:10.1371/journal.pone.0295061)
Supplement: S2 Table — (DOCX) [file pone.0295061.s005.docx]

|  | Univariable linear regression analysis | | | |
| --- | --- | --- | --- | --- |
|  | Length of OS (months) | | Length of PFS (months) | |
| Variable | β (95% CI) | p-value | β (95% CI) | p-value |
| ADAM22 | 16.94 (10.33 to 23.55) | < 0.001 | 13.78 (9.02 to 18.54) | < 0.001 |
| AEBP1 | –2.81 (–3.83 to –1.78) | <0.001 | –1.93 (–2.67 to –1.18) | <0.001 |
| ATP5C1 | 9.21 (6.08 to 12.33) | <0.001 | 5.59 (3.30 to 7.87) | <0.001 |
| C13orf18 | –3.38 (–4.71 to –2.05) | <0.001 | –2.74 (–3.69 to –1.78) | <0.001 |
| C1RL | –3.64 (–4.98 to –2.30) | <0.001 | –2.41 (–3.38 to –1.43) | <0.001 |
| CBR1 | –3.74 (–5.18 to –2.30) | <0.001 | –3.19 (–4.22 to –2.16) | <0.001 |
| CCL2 | –1.93 (–2.73 to –1.13) | < 0.001 | –1.39 (–1.97 to –0.81) | < 0.001 |
| CHI3L1 | –1.58 (–2.21 to –0.95) | <0.001 | –1.47 (–1.92 to –1.02) | <0.001 |
| CHL1 | –2.39 (–3.23 to –1.55) | <0.001 | –1.94 (–2.54 to –1.33) | <0.001 |
| CHST2 | –3.49 (–4.86 to –2.11) | <0.001 | –2.57 (–3.56 to –1.57) | <0.001 |
| CLEC5A | –3.01 (–4.17 to –1.84) | <0.001 | –2.34 (–3.18 to –1.49) | <0.001 |
| DHRS2 | 8.20 (5.59 to 10.80) | <0.001 | 4.79 (2.88 to 6.71) | <0.001 |
| DYNLT3 | –4.50 (–6.07 to –2.94) | < 0.001 | –3.98 (–5.10 to –2.87) | < 0.001 |
| EFEMP2 | –5.02 (–6.37 to –3.68) | <0.001 | –3.39 (–4.37 to –2.40) | <0.001 |
| EMP3 | –2.60 (–3.63 to –1.57) | <0.001 | –2.25 (–2.99 to –1.51) | <0.001 |
| F3 | –3.48 (–4.82 to –2.14) | <0.001 | –2.90 (–3.86 to –1.94) | <0.001 |
| FBXO17 | –4.72 (–6.49 to –2.96) | <0.001 | –3.46 (–4.74 to –2.18) | <0.001 |
| FLJ11286 | –4.25 (–5.91 to –2.59) | <0.001 | –3.23 (–4.43 to –2.03) | <0.001 |
| KIAA0495 | –8.66 (–5.91 to –2.59) | < 0.001 | –6.74 (–8.97 to –4.51) | < 0.001 |
| MSN | –4.73 (–6.31 to –3.14) | <0.001 | –3.42 (–4.57 to –2.27) | <0.001 |
| NSUN5 | –4.73 (–6.31 to –3.14) | <0.001 | –4.31 (–5.86 to –2.77) | <0.001 |
| PDPN | –2.32 (–3.23 to –1.42) | <0.001 | –1.89 (–2.54 to –1.24) | <0.001 |
| PGCP | –5.09 (–6.95 to –3.22) | <0.001 | –3.83 (–5.18 to –2.48) | <0.001 |
| PPCS | –5.15 (–7.23 to –3.06) | <0.001 | –4.26 (–5.76 to –2.75) | <0.001 |
| RAC3 | 8.47 (5.34 to 11.59) | < 0.001 | 5.63 (3.36 to 7.90) | < 0.001 |
| RANBP17 | 12.42 (7.86 to 16.99) | <0.001 | 8.85 (5.54 to 12.16) | <0.001 |
| RBP1 | –2.15 (–3.04 to –1.26) | <0.001 | –1.93 (–2.57 to –1.29) | <0.001 |
| SERPING1 | –2.80 (–3.85 to –1.75) | <0.001 | –1.86 (–2.63 to –1.09) | <0.001 |
| SHANK1 | 18.47 (11.34 to 25.60) | <0.001 | 13.38 (8.21 to 18.54) | <0.001 |
| SLC25A20 | –5.40 (–7.21 to –3.59) | <0.001 | –3.60 (–4.92 to –2.28) | <0.001 |
| SLC2A10 | –3.25 (–4.52 to –1.98) | <0.001 | –2.61 (–3.52 to –1.69) | <0.001 |
| STEAP3 | –3.17 (–4.47 to –1.86) | <0.001 | –2.88 (–3.82 to –1.95) | <0.001 |
| SWAP70 | –4.41 (–6.21 to –2.61) | <0.001 | –3.23 (–4.53 to –1.93) | <0.001 |
| TIMP1 | –3.13 (–4.35 to –1.90) | <0.001 | –2.82 (–3.69 to –1.94) | <0.001 |
| TMEM22 | –3.57 (–4.91 to –2.23) | <0.001 | –2.51 (–3.49 to –1.54) | <0.001 |
| TRIP6 | –3.72 (–5.25 to –2.20) | <0.001 | –2.70 (–3.80 to –1.59) | <0.001 |

OS: overall survival; PFS: progression-free survival; CI: confidence interval
